# Increased inflammation but similar physical composition and function in older-aged, HIV-1 infected subjects

**DOI:** 10.1186/s12865-015-0106-z

**Published:** 2015-07-24

**Authors:** Mark A. Wallet, Thomas W. Buford, Anna-Maria Joseph, Madhuri Sankuratri, Christiaan Leeuwenburgh, Marco Pahor, Todd Manini, John W. Sleasman, Maureen M. Goodenow

**Affiliations:** Department of Pathology, Immunology and Laboratory Medicine, University of Florida, Box 100275, Gainesville, FL 32610-0275 USA; Department of Aging and Geriatric Research, University of Florida, Gainesville, FL USA; Department of Medicine, Division of Infectious Diseases, University of Florida, Gainesville, FL USA; Department of Medicine, Division of Infectious Diseases, Malcom Randall VA Medical Center, Gainesville, FL USA; Department of Pediatrics, Division of Allergy and Immunology, Duke University, Durham, NC USA

## Abstract

**Background:**

Systemic immune activation (inflammation) and immunosenescence develop in some people with advancing age. This process, known as “inflamm-aging,” is associated with physical frailty and sarcopenia. Meanwhile, successful antiretroviral therapy has led to a growing number of older HIV-1-infected individuals who face both age-related and HIV-1-related inflammation, which may synergistically promote physical decline, including frailty and sarcopenia. The purpose of our study was to determine if inflammation during treated HIV-1 infection worsens physical impairment in older individuals.

**Methods:**

We determined the severity of HIV-associated inflammation and physical performance (strength and endurance) in 21 older HIV-infected individuals (54–69 years) receiving suppressive antiretroviral therapy, balanced for confounding variables including age, anthropometrics, and co-morbidities with 10 uninfected control individuals. Biomarkers for microbial translocation (lipopolysaccharide [LPS]), inflammation (soluble CD14 [sCD14], osteopontin, C-reactive protein [CRP], interleukin-6 [IL-6], soluble ICAM-1 [sICAM-1] and soluble VCAM-1 [sVCAM-1]), and coagulopathy (D-dimer) were assayed in plasma. Activation phenotypes of CD4^+^T cells, CD8^+^ T cells and monocytes were measured by flow cytometry. Physical performance was measured by 400 m walking speed, a short physical performance battery [SPPB], and lower extremity muscle strength and fatigue.

**Results:**

Overall physical function was similar in the uninfected and HIV-infected groups. Compared to uninfected individuals, the HIV-infected group had elevated levels of sCD14 (*P* < 0.001), CRP (*P* < 0.001) and IL-6 (*P* = 0.003) and an increased frequency of CD4^+^ and CD8^+^ T cells with an immunosenescent CD57^+^ phenotype (*P* = 0.004 and *P* = 0.043, respectively). Neither plasma inflammatory biomarkers nor CD57^+^ T cells correlated with CD4^+^ T cell counts. Furthermore, none of the elevated inflammatory biomarkers in the HIV-infected subjects were associated with any of the physical performance results.

**Conclusions:**

When age-related co-morbidities were carefully balanced between the uninfected and HIV-infected groups, no evidence of inflammation-associated physical impairment was detected. Despite careful balancing for age, BMI, medications and co-morbidities, the HIV-infected group still displayed evidence of significant chronic inflammation, including elevated sCD14, CRP, IL-6 and CD57^+^ T cells, although the magnitude of this inflammation was unrelated to physical impairment.

## Background

Antiretroviral treatment (ART) has dramatically increased the life expectancy of HIV-infected individuals. By 2015, more than one half of HIV-infected individuals are projected to be over 50 years old [1]. This increased life expectancy has prompted questions about how aging with HIV-1 infection combined with HIV treatments might interact to impact *active life expectancy (e.g.,* having adequate mobility to function as a member of the community).

HIV-1 infection causes systemic immune system activation fostered by a complex array of insults [2, 3]. The immune activation markers that are detected in HIV-1 infection are typically also elevated with increased age in the absence of HIV infection [3–5]. Similar to aging, HIV infection is associated with the premature development of cardiovascular disease, thromboembolic disease, type 2 diabetes, cancer, neurocognitive decline, end-organ disease and frailty even when effective ART is implemented [3, 5, 6].

In HIV infection, chronic stimulation by LPS and other substances emanating from a leaky intestinal barrier leads to the activation of innate immune cells, including monocytes and macrophages, which can be measured in sera based on the levels of the LPS-binding protein soluble CD14 [sCD14] [7–9], and sCD14 levels remain elevated even with prolonged therapy [10]. Notably, sCD14 has been implicated as a biomarker for the risk of non-AIDS mortality among HIV-infected subjects [11]. Additional markers of innate immune activation, including interleukin-6 [IL-6] and the acute phase molecule C-reactive protein [CRP], are also predictive of non-AIDS HIV mortality [12, 13]. Elevation of the coagulation factor D-dimer is independently associated with cardiovascular disease [14] and mortality in HIV infection [12]. The monocyte chemotactic protein osteopontin [OPN] is persistently elevated in HIV-1 infection [15] and is further increased in HIV-associated dementia [16]. Inter-cellular adhesion molecule-1 [ICAM-1] and vascular cell adhesion protein-1 [VCAM-1] are cleaved to their soluble forms following the activation of leukocytes or vascular endothelium. Both sICAM-1 and sVCAM-1 are elevated in HIV infection [17] and associated with endothelial activation and/or an increased risk of cardiovascular disease [18–21].

In addition to innate immune activation, adaptive immune activation is a hallmark of both aging and HIV-1 infection. Most notably, both advancing age and HIV-1 infection are associated with an increased frequency of memory CD4^+^CD45RA^−^CD45RO^+^ T cells as well as expanded CD4^+^CD57^+^ and CD8^+^CD57^+^ T cell populations. CD57-bearing T cells possess a unique inflammatory senescent phenotype, whereby the cells are able to produce large amounts of inflammatory cytokines such as TNF but demonstrate impaired proliferation [22, 23]. In both aging and HIV-1 infection, the direct cause of CD57^+^ T cell accumulation is unclear, although chronic viral replication, for example human cytomegalovirus, may drive the expansion of CD57 cells through immune attrition [24].

The proper structure (tissue composition) and function (strength & endurance) of the lower extremities is vital for maintaining mobility in late life. However, a paucity of research has investigated mobility functions and their determinants in HIV-infected older adults. Inflammation may provide a common link between HIV infection, aging and frailty. Both chronic HIV-1 infection (treated and untreated) and aging-associated frailty are characterized by elevated levels of pro-inflammatory cytokines such as TNF and IL-6 [12, 25–27]. How the effects of HIV-1 infection, aging and inflammation combine to affect physical function and body composition remain mostly unknown.

Here, we determined the magnitude of HIV-associated inflammation while accounting for the traditional risk factors for age-related illness. A key objective was to examine the relationships between biomarkers of inflammation and physical function and to understand whether HIV-1 infection compounds the relationship between chronic inflammation and functional decline in older individuals. The cohort included older HIV-infected individuals (average age = 59.7 years, range 54–69 years) undergoing suppressive ART, compared to non-infected participants who were closely balanced for confounding variables including age, anthropometrics (body mass index), co-morbidities (e.g., diabetes and cardiovascular disease) and smoking status.

## Methods

### Subject inclusion/exclusion and enrollment

Twenty-one HIV-infected participants were recruited from the Gainesville Veterans Administration HIV clinic and the community at large. All of the participants provided written informed consent based on documents approved by the University of Florida Institutional Review Board. The protocols at Malcom Randall VA Medical Center are approved concurrently through the University of Florida IRB. The inclusion criteria were as follows: age ≥ 54 years old, well-controlled HIV on ART for at least 12 months, CD4 > 100 cells/ul and plasma HIV RNA < 5,000 copies. Combination regimens were either NRTI-based (Efavirenz + FTC and tenofovir; one case was treated with Efavirenz + abacavir) or protease inhibitor (PI)-based (atazanavir, ritonavir, fosamprenavir, saquinavir or darunavir + FTC and tenofovir). At the time of the study, 20 HIV-infected subjects had plasma HIV RNA below 50 copies/ml (the cutoff for the assay); one subject had an HIV RNA level of 208 copies per ml. The CD4^+^ T cell counts in the HIV-infected subjects were typically above 500/mm^3^ (mean = 560, SD = 243). Subjects were excluded from the study for any of the following criteria: active AIDS-defining illness, taking stavudine or zidovudine, hepatitis B or C infection, severe arthritis, uncontrolled hypertension, unstable angina, severe congestive heart failure, low body mass index (<20 kg/m^2^), poorly controlled diabetes, treatment for cancer in the previous 6 months, peripheral vascular disease, Parkinson’s disease, multiple sclerosis, amyotrophic lateral sclerosis, renal failure, use of anabolic steroids, cognitive impairment identified as having a Mini-Mental State Exam Score < 24, or inflammatory disease (rheumatoid arthritis, inflammatory bowel disease, among others). It was expected that there would be increased inflammation in the HIV-infected group, and elevated sCD14 levels have been widely reported as a common feature of HIV-related inflammation [11]. With the goal of determining how inflammation relates to physical composition/function, we powered the study on plasma sCD14 levels. Ten HIV-uninfected control participants were recruited after screening the HIV-infected subjects for plasma levels of sCD14. The mean sCD14 level of 21 HIV-infected subjects was 1,892 ng/ml (SD = 394). Using α = 0.05, *n* = 9 provides 80 % power to detect a 25 % difference in sCD14 levels between the groups. HIV-infected subjects frequently develop age-related co-morbidities such as cardiovascular disease and respiratory disease, so a ‘healthy’ non-HIV infected control group would not be suitable for comparison. A self-report questionnaire was used to assess co-morbidities including cardiovascular conditions (controlled hypertension, previous hospitalization for myocardial infarction, pacemaker, stroke or abnormal heart rhythm) or respiratory conditions (shortness of breath, asthma or recent chest congestion). Control participants were recruited from the community, enrolled after testing negative for HIV, and balanced to the HIV cases based on average age, body mass index, and smoking status, as well as the frequency of active diabetes, cardiovascular conditions and pulmonary conditions (Table 1). Following the balancing approach, there were no significant differences between the groups in terms of age, BMI, chronic diseases or the use of non-HIV medications related to chronic disease.

**Table 1:** Cohort characteristics

	Uninfected (*n* = 10)	HIV-infected (*n* = 21)	*P* value
Age* years	62.5 (58–69)	59.7 (54–69)	0.100^a^
BMI* kg/m^2^	29.3 (24.8–39.5)	30.1 (20.7–71.8)	0.640^a^
Chronic disease			
Respiratory	30 %	33 %	1.000^b^
Cardiovascular	90 %	62 %	0.205^b^
Diabetes	30 %	38 %	1.000^b^
Current Smoker	20 %	24 %	1.000^b^
Non-HIV medications			
Aspirin regimen	50 %	43 %	1.000^b^
Hypertension	50 %	76 %	0.222^b^
Cholesterol	90 %	71 %	0.379^b^
Glucose control	30 %	28 %	1.000^b^
HIV medications			
NNRTI	N/A	57 %	N/A
NRTI	N/A	62 %	N/A
PI	N/A	33 %	N/A

*Mean (range), *N/A* not applicable; ^a^Mann-Whitney U-test, ^b^Fisher’s exact test

### Measurement of plasma biomarkers

Whole blood samples were collected in sterile Vacutainer™ (Becton Dickinson, Franklin Lakes, NJ) acid citrate dextrose tubes and processed within 12 h. The PBMC and plasma samples were stored at −180 °C in liquid nitrogen and −80 °C, respectively, in non-pyrogenic polypropylene cryovials (Nunc Cryotubes™) [28]. LPS levels were quantified using the Limulus Amebocyte Lysate [LAL] chromogenic assay (Lonza Inc., Allendale, NJ) as previously described [28]. The plasma samples were diluted 1:4 in 0.15 M NaCl prior to analysis, and the lower limit of detection was 0.1 endotoxin units [EU] per milliliter. The following soluble markers of immune and endothelial activation were measured by ELISA: sCD14, osteopontin [OPN], C-reactive protein [CRP], soluble ICAM-1 [sICAM-1], sVCAM-1 (R&D Systems Inc., Minneapolis, MN), and IL-6 (BD Biosciences, San Diego, CA). The coagulation marker D-dimer was measured by ELISA (American Diagnostica GmbH, Stamford, CT).

### Flow cytometry analysis of cell surface phenotypes

The following flow cytometry antibodies were purchased from BD Biosciences (San Jose, CA): anti-CD3-PE Cy7, anti-CD4-Alexa488, anti-CD8-PacBlu, anti-CD28-PE, anti-CD57-APC, anti-CD45RO-Alexa 700, anti-HLA-DR-APC, anti-CD14-PacBlu, anti-CD11a-FITC, anti-CD16-PE-Cy7, anti-CD163-PE, anti-CD62L-APC, and anti-CD86-Alexa 700. Two multi-color panels were used for T cells, and two panels were used for monocytes. Data were collected using a BD LSRII flow cytometer and analyzed with FCS Express software (DeNovo Software, Los Angeles, CA).

### 400 m rapid walk

Participants were asked to walk 400 m (20 m per lap) at a rapid pace as described elsewhere [29]. Walking speed was calculated as the distance walked divided by the time elapsed. At the end of each lap, the participants were asked about their physical exertion on a 0 (none) to 10 (highest) scale [30]. Lap variability may indicate fatigue and was calculated as the standard deviation in split times for each lap.

### Short physical performance battery (SPPB)

The SPPB test is a common measure of physical performance in older adults and is described elsewhere [31]. Briefly, the test consists of timed measures of standing balance in three positions (side by side position, semi tandem position, and tandem position), walking speed over 4 m, and time to stand up and sit down 5 times in a chair as quickly as possible. Each of the 3 performance measures was assigned a score ranging from 0 to 4 according to normative data published elsewhere [31], with 4 indicating the highest level of performance, and 0 representing an inability to complete the test. A summary score was created by adding each performance score; the summary score therefore ranges from 0 to 12. Excluding the balance test, values were reported for the speed (or time) to complete each task and score.

### Lower extremity tissue composition

T1-weighted 3D-magnetic resonance imaging (MRI) was used to quantify the tissue volumes of the right leg using a Phillips 3.0 Tesla magnet (Philips Medical Systems, Bothell, WA) as described previously [32]. Muscle, subcutaneous adipose tissue (SAT), and inter-muscular adipose tissue (IMAT) were measured volumetrically over 20 contiguous axial slices (10 in the mid-thigh and 10 in the mid-calf region) as previously described by our group [33]. Values are expressed as the absolute volume in centimeters cubed (cm^3^) and as a percent of the total volume. MRIs were collected for 18 HIV cases and 10 non-infected controls.

### Lower extremity muscle strength and fatigue

Maximal knee extension and flexion isokinetic peak torque were measured using a Biodex isokinetic dynamometer (Shirley, NY). Participants were asked to complete 50 concentric contractions at 90°/s with their right leg. The peak torque (in Newton-meters) and total work (in joules) achieved during the trials was used for the data analyses. A fatigue index was calculated as the change in muscle work (in joules) during the first 16 repetitions (the 1^st^ third) compared to the last 16 repetitions (the last 3^rd^). A negative value indicated a decrease in muscle work capacity in the final 16 repetitions.

### Statistical analyses

For all parameters, outliers were detected using the Grubb’s extreme studentized deviate [ESD] method with an alpha value set at 0.01. After the outliers were removed, each parameter was tested for a normal distribution using the D’Agostino & Pearson omnibus normality test. Normally distributed parameters were compared using unpaired t-tests, and non-normally distributed parameters were compared by Mann–Whitney U-tests. A total of 5 outlier values were detected and removed; when the outliers were re-introduced and the analyses were re-run, no qualitative differences in outcomes or new significant differences were detected. All of the reported data excluded outliers. Pearson’s correlation and simple linear regression were used to determine the relationships between two variables.

## Results

### Innate immune activation in older HIV-infected individuals

To determine the severity of innate immune activation, a panel of plasma biomarkers was assessed. LPS levels were similar in HIV-infected individuals and uninfected individuals (Table 2). Likewise, some markers of immune activation/coagulation, including OPN and D-dimer, were similar between the un-infected and infected groups. Trends toward elevated sICAM1 (*P* = 0.022) and sVCAM1 (*P* = 0.009) were observed, but neither factor reached the significance threshold of *P* = 0.006 (adjusted for multiple comparisons). There were, however, significantly higher levels of sCD14, CRP and IL-6 in the HIV-infected group compared to the uninfected group.

**Table 2:** Elevated inflammation biomarkers but similar microbial translocation in older HIV-infected subjects

	Uninfected *n* = 10	HIV *n* = 21	*P* value*
LPS EU/ml	0.37 (0.30/0.44)	0.34 (0.26/0.42)	0.638
sCD14 ng/ml	1154 (993/1315)	1892 (1716/2069)	≤0.001
OPN ng/ml	38.9 (32.8/44.9)	45.7 (37.4/54.0)	0.257
sICAM-1 ng/ml	631 (510/766)	1086 (815/136)	*0.022*
sVCAM-1 ng/ml	49.0 (34.5/63.6)	71.4 (61.6/81.2)	*0.009*
CRP ng/ml	650 (369/931)	4582 (2675/6488)	≤0.001
IL-6 pg/ml	0.42 (0.08/0.92)	2.10 (1.19/3.01)	0.003
D-dimer ng/ml	104.5 (4.2/204.8)	229.1 (72.6/385.6)	0.417

All values are means (5/95 % CI). *Mann–Whitney U-test. The underlined values are statistically significant at *P* < 0.006 (adjusted for multiple comparisons); the *Italicized* values show a trend toward statistical significance at *P* < 0.05

We then compared individual markers of immune activation to determine whether elevated levels of soluble factors were correlated or independently regulated. When a correlation matrix was applied, no association between plasma LPS and any soluble marker of inflammation was found (Table 3). When each inflammatory marker was compared to the panel of markers, only sVCAM1 and sICAM1 displayed the expected strong positive correlation (*r* = 0.700, *P* < 0.001).

**Table 3:** Correlations between plasma inflammation biomarkers

	sCD14	CRP	IL-6	sICAM1	sVCAM1
LPS	0.306	−0.077	−0.240	−0.070	−0.116
sCD14		0.395	−0.076	0.456	0.217
CRP			0.107	0.420	−0.052
IL-6				0.409	0.0364
sICAM1					*0.700*
sVCAM1					

Pearson correlation *r* values, *underlined* *= P* < 0.006 (corrected for multiple comparisons)

### Reduced monocyte frequency in HIV-infected subjects

**Fig. 1:**
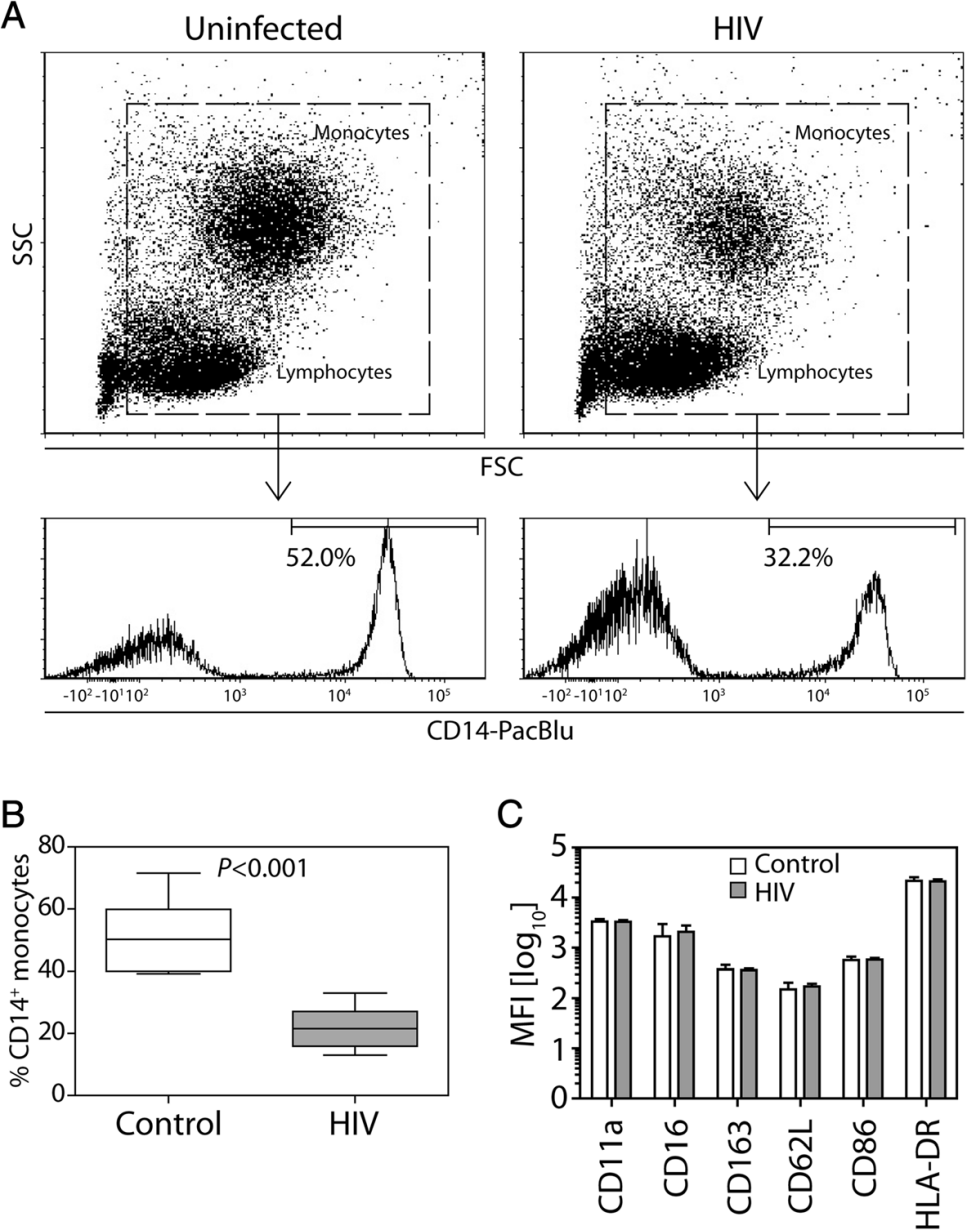
Reduced frequency of monocytes among PBMCs from HIV-infected subjects. **a** PBMC samples were analyzed by flow cytometry by first gating on the total mononuclear cells and then measuring the frequency of CD14^+^ cells for each individual (representative subjects). **b** The frequency of CD14^+^ monocytes among PBMCs from 21 HIV-infected subjects was compared to that of 10 uninfected controls using unpaired t-tests. **c** Using flow cytometry, the mean fluorescence intensity for a panel of monocyte maturation/activation markers was determined for CD14^+^ cells. No differences were found between the groups

Because HIV infection results in elevated plasma levels of sCD14, we sought to determine whether the circulating monocytes displayed an activated phenotype. Total PBMCs (lymphocytes and monocytes) were analyzed for the frequency of CD14^+^ monocytes in uninfected control and HIV-infected subjects (Fig. 1a). The frequency of CD14^+^ monocytes in the total PBMCs was significantly lower in HIV-infected subjects (Fig. 1b). Monocyte activation was measured based on the expression of the cell surface proteins CD11a, CD16, CD163, CD62L, CD86 and HLA-DR. Despite significantly elevated sCD14 levels in the plasma of HIV-infected subjects, there was no evidence that any of the activation markers differed in peripheral blood monocytes (Fig. 1c).

### T cell activation in HIV-infected subjects

**Fig. 2:**
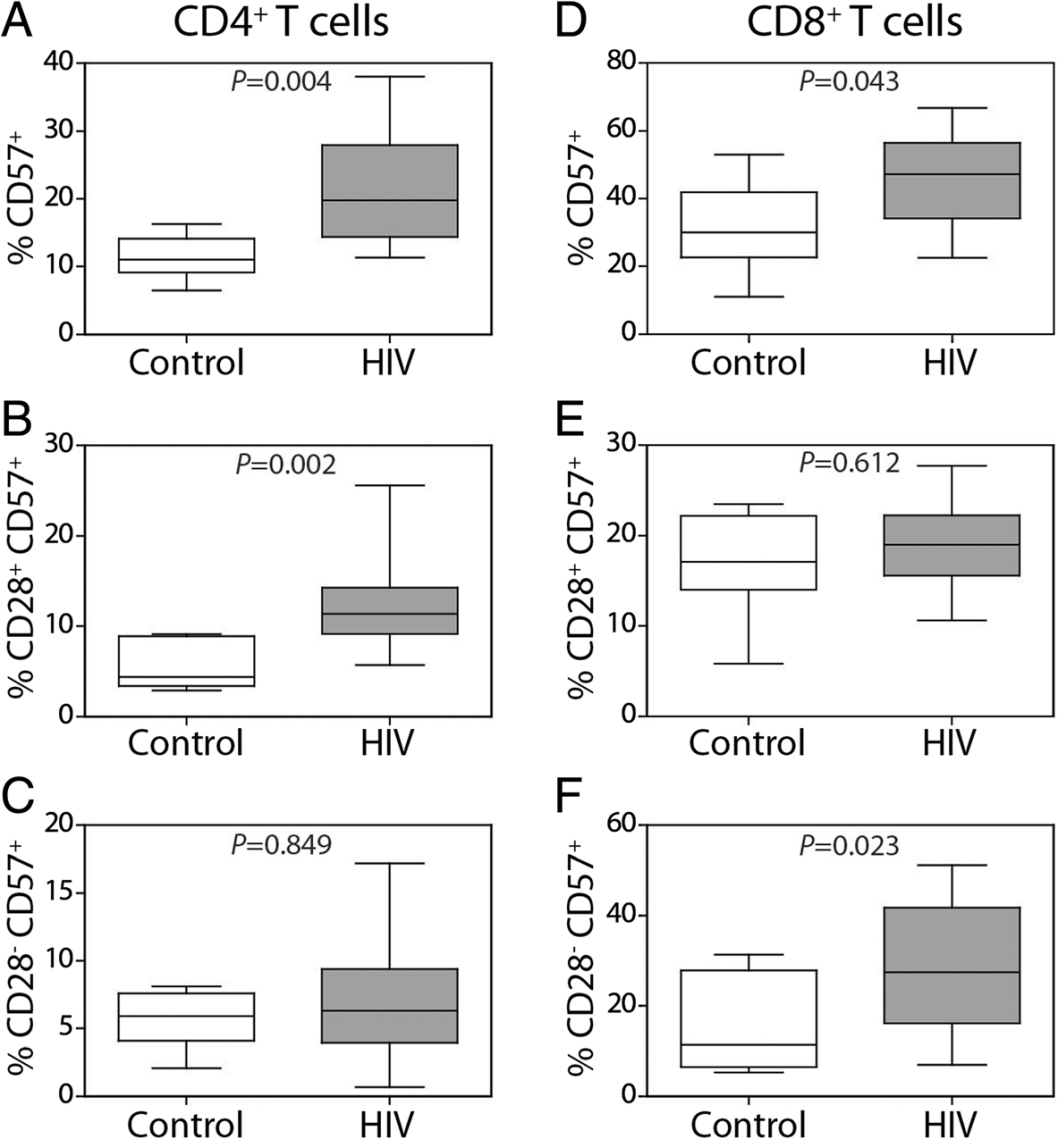
Increased frequency of CD57-expressing CD4^+^ and CD8^+^ T cells in HIV-infected subjects. PBMC samples were analyzed by flow cytometry by first gating on the total mononuclear cells and then on the CD3^+^ T cells. The frequency of CD57^+^ cells among the (**a**) total CD4^+^ T cells, (**b**) CD28-expressing CD4^+^ T cells or (**c**) CD28-negative CD4^+^ T cells was determined. Next, the frequency of CD57^+^ cells among the (**d**) total CD8^+^ T cells, (**e**) CD28-expressing CD8^+^ T cells or (**f**) CD28-negative CD8^+^ T cells was determined using Mann–Whitney U-tests. The error bars represent ± 1 SD

T cell senescence is characterized by the loss of CD28 expression and the increased expression of CD57 in CD4 or CD8 T cells [23, 34]. We found that in older HIV-infected subjects, the frequency of CD57^+^ T cells (both CD4^+^ and CD8^+^) was significantly elevated in comparison to uninfected subjects (Fig. 2a and d). The CD57^+^ cells were further classified based on the expression of CD28. Among CD4^+^ cells, the CD57^+^ CD28^+^ subset accounted for the difference observed in HIV-infected subjects (*P* = 0.002) (Fig. 2b) whereas no difference in the CD4^+^ CD57^+^ CD28^−^ population was found between uninfected and HIV-infected subjects (*P* = 0.849) (Fig. 2c). In contrast, among CD8^+^ T cells, the CD57^+^ CD28^+^ populations were similar between the uninfected and HIV groups (*P* = 0.612) (Fig. 2e), but a higher frequency of CD8^+^ CD57^+^ CD28^−^ T cells was observed in the HIV-infected group (*P* = 0.023) (Fig. 2f). The frequencies of CD4^+^ CD57^+^ CD28^+^ and CD8^+^ CD57^+^ CD28^−^ cells were positively correlated in the HIV-infected subjects (*r* = 0.512, *P* = 0.025) (data not shown).

**Fig. 3:**
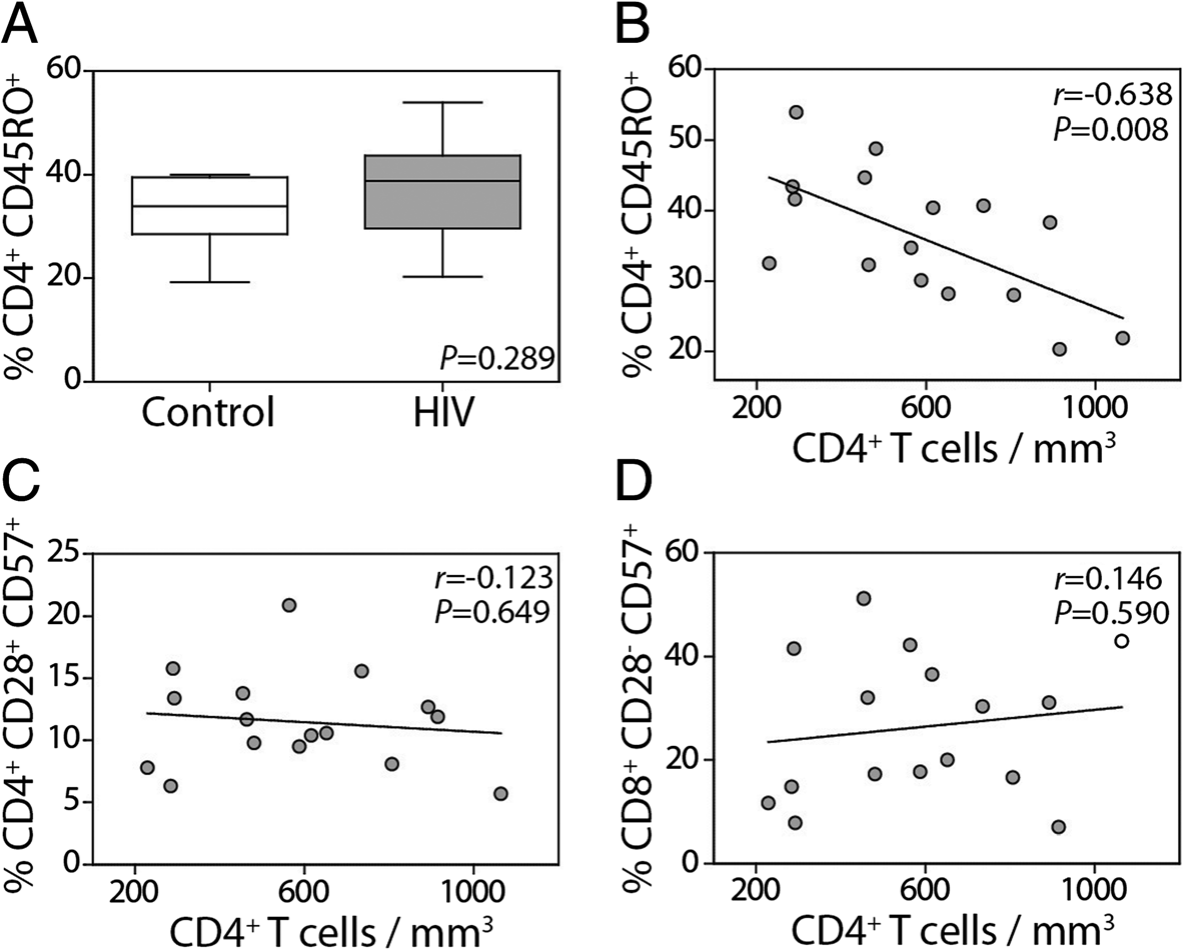
Frequency of CD45RO-expressing memory CD4^+^ T cells, but not CD57^+^ CD4^+^ T cells, is associated with CD4^+^ T cell decline. PBMC samples were analyzed by flow cytometry by first gating on the total mononuclear cells and then on the CD3^+^ T cells. The frequency of (**a**) CD4^+^ CD45RO^+^ T cells was then determined. The correlations between total peripheral blood CD4^+^ T cell counts and (**b**) T cells CD4^+^ CD45RO^+^ T cells, (**c**) CD4^+^ CD28^+^ CD57^+^ T cells, or (**d**) CD8^+^ CD28^−^ CD57^+^ T cells were determined (Pearson correlation)

One of the strongest correlates for CD4^+^ T cell decline in HIV infection is the increased frequency of memory/effector CD4^+^ CD45RO^+^ T cells and a corresponding deficit in CD4^+^ CD45RA^+^ naïve T cells [28, 35, 36]. In this cohort of older ART-treated HIV subjects, there was no difference in the frequency of CD4^+^ CD45RO^+^ T cells between the groups (Fig. 3a), yet the frequency of these memory/effector cells was inversely correlated with the peripheral blood CD4^+^ T cell counts in the HIV-infected individuals (*r* = −0.638, *P* = 0.008) (Fig. 3b). Neither the CD4^+^ CD57^+^ CD28^+^ nor CD8^+^ CD57^+^ CD28^-^ T cell frequency was associated with the CD4^+^ T cell counts (Fig. 3c and d).

### Physical composition and performance

Lower extremity tissue composition measurements were available for 18 of the 21 HIV-positive participants (Table 4). There were no significant differences in leg tissue volumes, and the measures of subcutaneous and intramuscular adiposity were similar between the HIV and control groups. Muscle strength and fatigue were similar between the control and HIV-infected groups, with no difference in knee extension peak torque, knee flexion peak torque, knee extension total work, knee flexion total work, knee extension fatigue index or knee flexion fatigue index.

**Table 4:** Physical composition and performance

	Control (*N* = 10)	HIV (*n* = 21)	*P*-value
Leg tissue volume (values in cm^3^) ^a^			
Total circumference	651 (170)	637 (170)	0.841
Muscle tissue	353 (69.6)	336 (52.8)	0.483
Subcutaneous adipose tissue	185 (105)	155 (116)	0.512
Inter-muscular adipose tissue	97.2 (25.8)	102 (26.2)	0.601
Leg tissue percent of total circumference ^a^			
Muscle tissue	54.8 (4.2)	54.3 (8.8)	0.875
Subcutaneous adipose tissue	28.0 (14.3)	22.6 (10.0)	0.259
Inter-muscular adipose tissue	15.1 (2.7)	16.4 (3.3)	0.315
Thigh muscle strength & fatigue			
Knee extension peak torque (Nm)	96.4 (40.1)	103.9 (30.4)	0.562
Knee flexion peak torque (Nm)	50.7 (22.3)	53.7 (20.0)	0.705
Knee extension total work (joules)	3039 (1453)	3396 (967)	0.422
Knee flexion total work (joules)	1552 (800)	1561 (744)	0.977
Knee extension fatigue index (joules)	−704 (600)	−781 (581)	0.735
Knee flexion fatigue index (joules)	−288 (355)	−322 (294)	0.784
SPPB			
4-meter gait speed (m/sec)	1.11 (0.10)	1.0 (0.18)	0.102
4-meter gait speed score (range: 0–4)	4 (0)	3.8 (0.51)	0.253
Time to complete 5 chair stands (sec)	10.2 (2.1)	12.1 (2.4)	0.047
Chair stand score (range: 0–4)	3.6 (0.51)	3.0 (0.97)	0.105
Balance score (range: 0–4)	4 (0)	3.85 (0.65)	0.499
Total performance score (range: 0–12)	11.6 (0.51)	10.7 (1.9)	0.167
400-meter rapid walk			
400-meter rapid gait speed (m/sec)	1.42 (0.16)	1.28 (0.24)	0.126
Average exertion (range: 0–10)	1.47 (1.70)	1.14 (1.04)	0.585
Maximal exertion (range: 0–10)	2.69 (2.89)	2.03 (1.52)	0.502
Lap variability in gait speed (m/sec)	2.07 (4.21)	1.08 (0.48)	0.465

Values are means (SD) for continuous variables unless otherwise indicated

*Nm* Newton-meters

*cm* ^*3*^ cubic centimeters

^a^ Tissue volumes collected in 10 non-infected controls and 18 HIV cases

The average SPPB score for the control group was 11.6 (0.51), whereas the HIV group demonstrated a score of 10.7 (1.9) (*p* = 0.167). When the components of the performance battery were examined separately, the HIV-infected subjects required more time to complete five chair stands compared to the uninfected controls (12.1 s vs 10.2 s, *P* = 0.047), but otherwise both groups were similar across the individual components of the battery. Finally, both the control and HIV groups showed similar performances in the rapid walking test, with no significant differences in 400-meter gait speed, average exertion or maximal exertion. No differences were observed for peak knee extension and flexion or total knee extension and flexion. Overall, the physical performance of the control and HIV-infected groups was remarkably similar. Across all subjects and within HIV+ and control groups, exhaustive correlation analyses were performed to compare each HIV-associated inflammatory biomarker (sCD14, CRL, IL-6, CD57^+^ T cells) with each measure of physical composition and performance. No significant associations were detected for any of the comparisons (data not shown). Thus, inflammation associated with HIV is not related to physical composition or function in this cohort of older-aged individuals.

## Discussion

Both HIV infection and normal aging in the absence of HIV-1 infection are associated with chronic inflammation that negatively impacts overall health. Frailty develops earlier in HIV-infected individuals than uninfected individuals [37, 38], and a suspected source of increased frailty is chronic inflammation which develops early in HIV-1 infection [39]. In non-HIV-1-infected older persons, there is a well-established correlation between the biomarkers IL-6, TNF and CRP and frailty, as demonstrated in the Newcastle 85+ study [40], although no association between immunosenescence and frailty was detected. In HIV-1 infection, the VACS index, a multi-parameter score that combines biomarkers for HIV-1 disease and organ system injury but not inflammation, predicts frailty [41]. Here, we investigated how HIV infection augments the inflammation that occurs normally in older individuals with the goal of understanding whether potentially additive inflammatory effects of HIV-1 and advancing age accelerate the development of frailty. Our understanding of the effects of HIV-1 and advancing age on systemic inflammation may be confounded by additional factors including obesity, age-related co-morbidities, behaviors (e.g., smoking) and non-HIV medications. We carefully balanced HIV-1-infected and un-infected subjects for these factors and found that HIV-1 infection, even with suppressive antiretroviral therapy, is associated with a number of unique inflammatory phenotypes compared to a control group with similar co-morbidity and medication profiles. As a population, the HIV-1-infected group had significantly elevated levels of plasma sCD14, CRP and IL-6 compared to the uninfected controls. Although they failed to reach statistical significance, modestly elevated levels of sICAM1 and sVCAM1, markers of endothelial activation, were observed in the HIV-infected subjects. Our previous studies in infected and uninfected young adults revealed similar differences in sVCAM levels [42].

Based on our previous work and that of others [7, 9, 28, 43–47], we hypothesized that plasma LPS levels would be elevated in older HIV-infected subjects compared to uninfected subjects, however, LPS levels were similar in both groups. Our previous studies of microbial translocation focused on much younger individuals (infants and children), among whom there were clear differences in LPS levels in HIV-1-infected children compared to uninfected children [28]. The similar levels of plasma LPS among the uninfected and HIV-1-infected older individuals was surprising, but we can speculate that there may be age-related differences in intestinal permeability and microbial translocation (even in the absence of HIV-1 infection) that mask the biomarkers of gut pathology that are easily detected in younger cohorts. Alternatively, co-morbidities such as diabetes, heart disease or kidney disease, which were prevalent among both the HIV-infected and uninfected groups, may be associated with microbial translocation. For example, endotoxemia is associated with atherosclerosis in non-HIV-infected subjects [48].

Elevated levels of plasma sCD14 are clearly associated with poorer health status and health outcomes in HIV patients [7, 11, 49]. While the source of sCD14 is attributed to systemic monocyte/macrophage activation, there is little evidence to implicate peripheral blood monocytes versus tissue macrophages in the elevated production of sCD14. One recent study in younger subjects (median age = 41 years) found a positive correlation between plasma levels of IL-6, D-dimer, CRP, or sCD163 and multiple phenotypic alterations in peripheral blood monocytes, including the frequency of CD16^+^ monocytes [50]. We found no evidence of monocyte phenotypic alterations in HIV-infected subjects despite markedly elevated plasma levels of sCD14. We did find, however, that HIV-infected subjects had a reduced frequency of peripheral blood monocytes compared to uninfected subjects. Whether the association between high sCD14 and a low frequency of circulating monocytes is due to an increased rate of monocyte extravasation or apoptosis or alternatively to reduced production from bone marrow precursors remains unknown. Our previous study showed that human peripheral blood monocytes produce relatively higher levels of sCD14 in comparison to macrophages, yet only macrophages responded to LPS by releasing more sCD14 [51]. Together, these findings suggest that elevated sCD14 in older individuals may originate from tissue macrophages rather than monocytes.

The expansion of CD57-expressing T cells is typical of both HIV infection and advancing age [34]. In CD57^+^ cells specific for HIV antigens, replicative senescence arises from chronic antigenic exposure; this effect is similar to that observed in chronic infection with cytomegalovirus (CMV) associated with an increased frequency of both CD4^+^ and CD8^+^T cells expressing CD57 [52]. In healthy populations, the proportion of CD8^+^CD57^+^T cells expands with increasing age [53]. The combined effects of age and CMV infection result in an accumulation of CD8^+^ CD57^+^ T cells lacking the co-stimulatory molecule CD28 [54]. We observed a significant increase in the proportion of CD8^+^ CD28^−^ CD57^+^ but not CD8^+^ CD28^+^ CD57^+^ T cells in the HIV cohort. The CMV status of both HIV-infected and control groups was unknown, so the cause of expanded senescent CD8^+^ T cells requires further investigation.

Similar to CD8^+^ T cells, the expansion of CD4^+^ CD57^+^ CD28^−^ T cells is associated with chronic viral infections [55–57]. We found an increase in the frequency of CD4^+^ CD57^+^ CD28^+^ T cells in HIV-infected subjects but no difference in CD4^+^ CD57^+^ CD28^−^ T cells. Expansion of CD57-expressing T cells occurred independently of the total CD4 T cell number in HIV-infected subjects. Instead, the increased frequency of memory CD4^+^ CD45RO^+^ T cells was associated with decreased CD4^+^ T cell numbers. This finding is in accordance with another study showing that increasing age and the concomitant reduced production of naïve CD4^+^ T cells, rather than the expansion of senescent CD57^+^ T cells, underlies the deficit in CD4 T cell reconstitution observed in younger, treated HIV patients [24].

We anticipated that HIV disease would result in some degree of frailty and/or reduced physical performance compared to similarly-aged uninfected control subjects. Frailty is a common trait of HIV-1 infection and has been linked to numerous causes including chronic inflammation, polypharmacy, and coagulopathy [39, 58]. Here, the control and HIV groups demonstrated nearly identical physical characteristics with similar leg tissue volume and adiposity. Strength, fitness and fatigue measures were also highly similar between the groups. Frailty in HIV-1 infection is strongly correlated with a high viral load and CD4^+^ T cell decline [38, 41], whereas lipodystrophy syndrome and fat redistribution are associated with combination antiretroviral therapy [59]. The patients in our study had well-controlled viremia and stable CD4^+^ T cell counts, which may in part explain the similarities in leg musculature between the uninfected and infected groups. It is well established that antiretroviral therapy can cause redistribution of limb fat to the trunk [59] or increased visceral fat within the limbs [60], but no change in leg adiposity was observed in our HIV-1-infected group. It is possible that the variability in measurable lipodystrophy in the HIV-1 infected individuals (estimates range from 13 to 70 % [59]) is too great to observe changes on our relatively small cohort. In addition, exclusion of subjects taking the NRTI drugs stavudine or zidovudine may explain the similarities in adiposity, because HIV-associated lipoatrophy has been related to exposure to these drugs [61].

Despite strong evidence for increased inflammation in the HIV-1 group, the infected and uninfected groups had similar muscle and physical performance characteristics. Thus, although inflammation is a characteristic of aging-related frailty and HIV-1 infection, our study suggests that increased inflammation in older HIV-1-infected individuals causes no greater frailty than what is found in similarly aged uninfected individuals. Ours is the first study that we know of where older uninfected and HIV-1-infected individuals were carefully balanced for age-related co-morbidities and medications to specifically assess the role of HIV-associated inflammation in frailty. A major limitation of our conclusion is that the study size is small; this work should be repeated in larger studies. Nonetheless, inflammation was profoundly elevated in the HIV-1-infected group, so we accept this as convincing evidence that inflammation alone does not always lead to frailty.

## Conclusions

HIV infection is thought to augment the inflammatory consequences of advancing age, with many of the health challenges of old age being faced by far younger infected adults. However, HIV infection is no longer a disease of youth. Improvements in therapy are resulting in a predictable “aging up” of the HIV-infected population. In the state of Florida, the location of our current study, the percentage of total HIV cases among people over the age of 50 years increased from 15 % in 2007 to 23 % in 2011 [62, 63]. As new interventions are sought to optimize the long-term care of aging HIV patients, it is important to appreciate the specific causes of morbidity/mortality that impact quality and length of life. Frailty is a characteristic of normal aging, and this process is accelerated in HIV-1 infection. It has been speculated that the common denominator between age-associated and HIV-associated frailty is excessive inflammation. Our study argues that HIV-1-associated inflammation may not lead to any greater frailty than what would be found in similarly-aged uninfected individuals when controlling for other health problems. Understanding the specific mechanisms that underlie age-related illnesses in older HIV-1-infected individuals will be of greater importance in the future as this population ages.

## Abbreviations

HIV-1: Human immunodeficiency virus type 1
LPS: Lipopolysaccharide
sCD14: Soluble CD14
CRP: C-reactive protein
IL-6: Interleukin 6
ICAM-1: Intercellular adhesion molecule 1
VCAM-1: Vascular cell adhesion protein 1
SPPB: Short physical performance battery
ART: Antiretroviral therapy
LAL: Limulus amebocyte lysate assay
MRI: Magnetic resonance imaging
SAT: Subcutaneous adipose tissue
IMAT: Inter-muscular adipose tissue
HNK1: Human natural killer 1
